# Are the Morphological Indices of the Vertebrobasilar System Heritable? A Twin Study Based on 3D Reconstructed Models

**DOI:** 10.3390/medicina57020127

**Published:** 2021-02-01

**Authors:** Laszlo Szalontai, Zsofia Jokkel, Tamas Horvath, Marton Piroska, Bianka Forgo, Csaba Olah, Laszlo Kostyal, David L. Tarnoki, Adam D. Tarnoki

**Affiliations:** 1 Medical Imaging Centre, Semmelweis University, 78/A Üllői Street, 1082 Budapest, Hungary; zsofijokkel@gmail.com (Z.J.); piroskamarton94@gmail.com (M.P.); tarnoki4@gmail.com (D.L.T.); tarnoki2@gmail.com (A.D.T.); 2 Oncologic Imaging Diagnostic Center, Department of Radiology, National Institute of Oncology, Ráth György Street 7-9, 1122 Budapest, Hungary; 3 HeartBit, Hűvösvölgyi Road 42, 1021 Budapest, Hungary; tom.horvath.md@gmail.com; 4 Department of Radiology, Faculty of Medicine and Health, Örebro University, Södra Grev Rosengatan, 701 85 Örebro, Sweden; fbia021@gmail.com; 5 Department of Neurosurgery, University of Debrecen, Nagyerdei Boulevard 98, 4032 Debrecen, Hungary; olahcs@gmail.com; 6 Department of Radiology, Borsod County University Teaching Hospital, 72-76 Szentpéteri Gate, 3526 Miskolc, Hungary; kostyalfed@gmail.com

**Keywords:** twin study, heritability, basilar artery curve, vertebral artery dominance, 3D vascular reconstruction

## Abstract

*Background and Objectives*: The asymmetrical vertebral artery (VA) flow and diameter are common findings, which can result in an asymmetrical blood flow in the basilar artery (BA), leading to bending of the artery over time. This study investigated whether the variation of the different vertebrobasilar morphological indices that influence flow characteristics might be inherited. *Materials and Methods:* We analyzed 200 cerebral magnetic resonance imaging (MRI) scans of healthy Caucasian twins (100 pairs) who underwent time-of-flight MRI. From the scans, we reconstructed the 3D mesh of the posterior circulation from the start of the V4 segment to the basilar tip and subsequently analyzed the morphology of the vertebrobasilar system. The phenotypic covariances of the different morphological parameters were decomposed into heritability (A), shared (C), and unshared (E) environmental effects. *Results:* 39% of the twins had left dominant VA, while 32.5% had right dominant. In addition, 28.5% were classified as equal. The vertebral artery V4 segment diameter, curvature, and tortuosity were mainly influenced by shared (C) and unshared (E) environmental factors. A moderate heritability was found for the BA length (A: 63%; 95% CI: 45.7–75.2%; E: 37%; 95% CI: 24.8–54.3%) and volume (A: 60.1%; 95% CI: 42.4–73.2%; E: 39.9%; 95% CI: 26.8–57.6%), while the torsion of both arteries showed no heritability and were only influenced by the unshared environment. *Conclusions:* The length and volume of the BA show a moderate genetical influence. However, most of the measured morphological indices were influenced by shared and unshared factors, which highlight the role of the ever-changing hemodynamic influences shaping the geometry of the vertebrobasilar system.

## 1. Introduction

**Figure 1:**
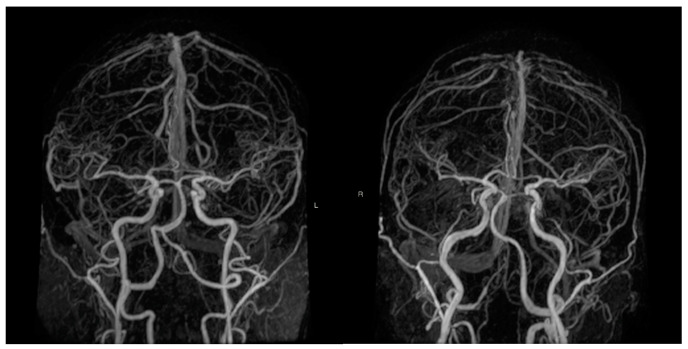
Magnetic resonance angiography images of two 76 year-old dizygotic twins. Right dominant vertebral artery and a left leaning basilar artery (left picture). Left dominant vertebral artery and a right leaning basilar artery (right picture). (Source: Medical Imaging Centre, Semmelweis University, Budapest, Hungary).

A stroke remains the second most common cause of death in the European Union, and it is the leading cause of disability amongst adults. A stroke leads to 440,000 deaths and affects 1.1 million citizens yearly [1]. Approximately 20% of all strokes occur in the posterior region of the brain, which is supplied by the vertebrobasilar system, with high mortality and morbidity [2]. The vertebrobasilar system is unique in anatomical terms, as it is the only instance in the human body where a third artery is formed by the merging of two arteries. The asymmetrical vertebral artery flow and diameter are common findings, and the vertebral system is often left dominant. This can result in an asymmetrical blood flow in the basilar artery, which may cause bending of the artery over time (Figure 1). Increased vertebral artery (VA) difference, tortuosity and torsion as well as increased basilar artery (BA) curvature and tortuosity may cause a turbulent blood flow, which plays an important role in the pathogenesis of atherosclerosis [3]. Furthermore, it has been shown that the posterior circulation is much more susceptible to atherosclerosis in comparison with the anterior circulation [4]. These vertebrobasilar morphological indices have also been linked to peri-vertebrobasilar infarcts as risk factors and have indicated a directional and locational correlation [3,5,6,7].

Although the relationship between these vertebrobasilar morphological indices has been associated with a stroke and atherosclerotic plaque localization, the understanding of the genetic and environmental determinants influencing the development of these indices are not yet clear. Our research group has already investigated the VA difference and heritability [8], however, that study utilized an ultrasound imaging technique and only investigated the V1 and V2 segments of the vessel. In this study, we reconstructed the vertebrobasilar system of healthy twins based on time-of-flight magnetic resonance imaging (TOF-MRI) sequences with a semi-automated segmentation software. To maintain objectivity, we created an in-house, semi-automated measuring software with Python scripts to accurately measure the morphological indices of the reconstructed models. Our hypothesis was that most of the measured parameters are influenced by genetic factors, which we based on our previous findings.

## 2. Materials and Methods

Two hundred healthy Caucasian twins (100 pairs) were randomly selected for our study from the Hungarian Twin Registry [9]. The study protocol conforms to the ethical guidelines of the 1975 Declaration of Helsinki and the study protocol was approved by the local Ethical Committees (Semmelweis University TUKEB 189-1/2014, BAZM Hospital Ethical Committee approved on 06 October 2016). All the participants signed an informed consent form. Self-reported questionnaires were used to maximize the accuracy of zygosity classification and to collect a detailed medical history and risk factors [10]. Exclusion criteria consisted of pregnancy, claustrophobia, previous surgery or occlusion of the carotid artery, or intervention in the vertebrobasilar system.

We recorded exercise, smoking, alcohol consumption, body weight, height, body mass index (BMI), hypertension, diabetes, and hyperlipidemia. Former and current smokers were included in the smoking group.

**Figure 2:**
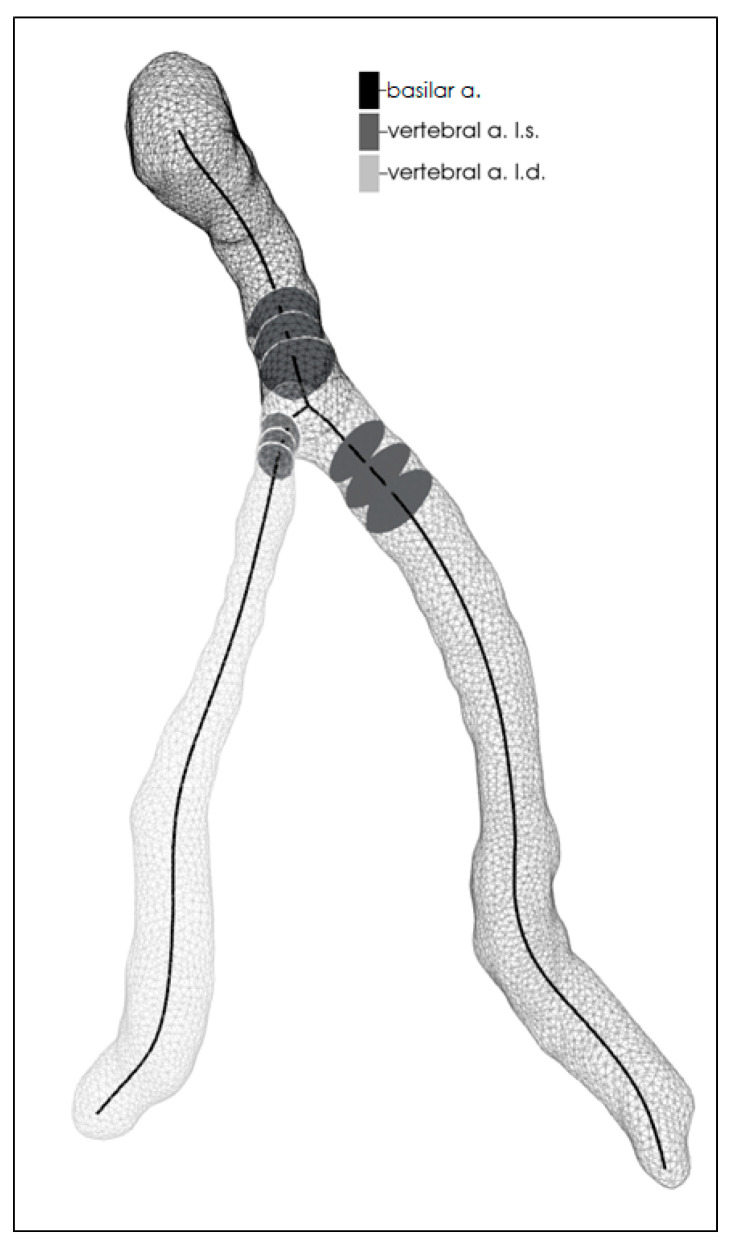
Visual representation of our in-house written software analyzing the vertebrobasilar reconstruction of a patient. The program identifies the arteries based on the confluence of the two vertebral arteries. Next, the program creates the centerline for each vessel and measures the different geometrical indices.

Each twin underwent a 3D time-of-flight magnetic resonance imaging (TOF MRI) of the vertebrobasilar system (Philips Ingenia 1.5 T). We created a 3D reconstruction of the vertebrobasilar system for each twin based on the TOF MR images. We used the ITK-SNAP software’s (version 3.8.0.) built in semi-automated segmentation tool, which is currently considered the gold standard for modeling arteries. After setting a lower (approx. 850 ± 50 gray level intensity) and an upper (approx. 2500 gray level intensity) threshold and placing markers to indicate where the different arteries start and end, the program automatically constructs the model. The disadvantage of this method is that artifacts and unwanted, smaller side-branches may appear during the reconstruction process. To eliminate this, each model was smoothed with the Taubin algorithm and unwanted branches were sliced off using MeshLab (v2016.12), thus obtaining the same three main arteries to ensure a standardized measurement. The descriptors of the vertebrobasilar geometry were extracted from the smoothed meshes semi-automatically by using VMTK (vascular modelling toolkit) scripts within a Python environment [11,12]. After placing markers at the start of the vertebral arteries and at the basilar tip, the VMTK-based scripts can recognize the left and right vertebral arteries along with the basilar artery and provide standardized measurements to analyze the morphological properties of these vessels (Figure 2). To eliminate artifact-based differences and to identify the dominant vertebral artery, a difference higher than 0.3 mm was set based on earlier publications. The cross-sectional area, curvature, torsion, and tortuosity were measured on both vertebral arteries. The VA curvature was defined as the inverse of the radius of the local osculating circle along the centerline of the vessel and VA torsion as the amount by which the osculating plane rotates along the centerline. VA tortuosity was defined as the shortest distance between the start and the end of the vessel divided by the actual centerline length of the vessel. On the basilar artery, we measured the length, volume, curvature, and cross-sectional area. The extent of the left or right basilar artery curvature was measured with a 2-step process. First, we connected the confluence of the two vertebral arteries and the basilar tip with a straight line. Second, we measured the median deviation from this straight line on the X axis. The BA torsion and tortuosity was calculated with the same method described previously.

### Statistical Analysis

A descriptive analysis (mean, standard deviation, and percentages) for the risk factors and vertebrobasilar parameters was calculated with SPSS Statistics v2.4. The classical twin study analyzes both monozygotic (MZ) and dizygotic (DZ) twin pairs at the same time. Higher levels of the intra-pair correlation between MZ pairs compared to DZ twins indicate a greater genetic influence on a phenotype, while a greater cross MZ and DZ twin similarity suggests that the variance is due to shared environmental factors. Similarly, larger levels of the pair correlation between DZ pairs compared to MZ twins indicate that the variance is influenced by unshared environmental components. It is known that identical twins share nearly 100% of their genome, while fraternal or non-identical twins (DZ) share roughly 50% of their genome in respect to a given phenotype. It is also known that both MZ and DZ twins share their common environment. The last portion of the variance, which shows a lower correlation in MZ twins compared to DZ twins, can be explained with the influence of the unique environment. Based on these principals, the univariate genetic modeling was performed with the RStudio version 1.3.1093 with OpenMx 2.18. The phenotypic variance of the different morphological parameters was decomposed into heritability (A), shared (C), and unshared (E) environmental effects (ACE analysis). A heritability estimate has been calculated using the within-pair correlation between MZ and DZ twins with 95% confidence intervals (CI). To investigate whether the anthropometric or cardiovascular risk factors influence the morphological indices beyond age and sex, bivariate regression analyses were performed. Based on these regression models, covariates have been added to further adjust the heritability models (Model 1 and Model 2). For each morphological phenotype, Model 1 only adjusts for age and sex, while Model 2 additionally corrects for all the risk factors with a significant relationship. The best fitting ACE models were chosen based on the Akaike and Bayesian information criterion (AIC and BIC).

## 3. Results

Of the 200 twins (100 pairs), 134 were monozygotic (67 MZ pairs) and 66 were dizygotic (33 DZ pairs). The average age was 49.6 (SD: ±14.4) and 56.0 (SD: ±15.2) years in the MZ and DZ groups, respectively. The two groups were significantly different in age (*p* = 0.004). The male to female ratio was 44:90 (67% female) in the MZ group and 23:43 (65% female) in the DZ group. No significant difference was observed between the MZ and DZ groups regarding anthropometric variables and risk factors. Table 1 shows the risk factors and the measured characteristics of our population.

Table 2 shows age- and sex-adjusted parameter estimates for additive genetic (A), common environmental (C), and unique environmental influences (E) on the different measured parameters by structural equation modeling. The within-pair correlation in monozygotic twins was higher than in dizygotics for the BA length (0.616 vs. 0.288) and BA volume (0.646 vs. 0.016). The age- and sex-adjusted additive genetic effect, within the most parsimonious model, accounted for 63% (95% CI: 45.7–75.2%) of the variance of the BA length and 60.1% (95% CI: 42.4–73.2%) of the variance of the BA volume. Unshared environmental effects accounted for 37% (95% CI: 24.8–54.3%) of the variance of the BA length and 39.9% (26.8–57.6%) of the variance of the BA volume. Although the within-pair correlation was higher in monozygotic twins than dizygotics both for the VA diameter difference and for the left and right VA tortuosity, the ACE model differed significantly from the saturated model, therefore, these results are only informative. The right VA curvature was moderately influenced by additive genetic factors (21%; 95% CI: 0–42.1%), and it was highly determined by the unshared environment (78.4%; 95% CI: 57.2–100%). No heritability was found for the rest of the measured parameters. There was no within-pair correlation for either monozygotic and dizygotic twins for the left and right VA torsion, therefore, the majority of the variances can be attributed to unshared environmental effects.

The final ACE models were corrected for age, sex, sport activity, alcohol, smoking, diabetes, hypertension, dyslipidemia, height, weight, and BMI. We constructed models where we only corrected for age and sex and compared the two types of models for all the examined variables. Table 3 shows the variables where there was a significant difference between the models.

From the fitted ACE models, we identified several significant environmental variables in our models, and two of them (smoking, height) were consistent across similar variables, therefore, we could conclude that smoking and height have a significant effect on the dimensions of the basilar artery, and smoking may also affect the vertebral artery (we could only fit the ACE model on the left vertebral artery, therefore, we do not have regression data about the right side) (Table 4).

**Figure 3:**
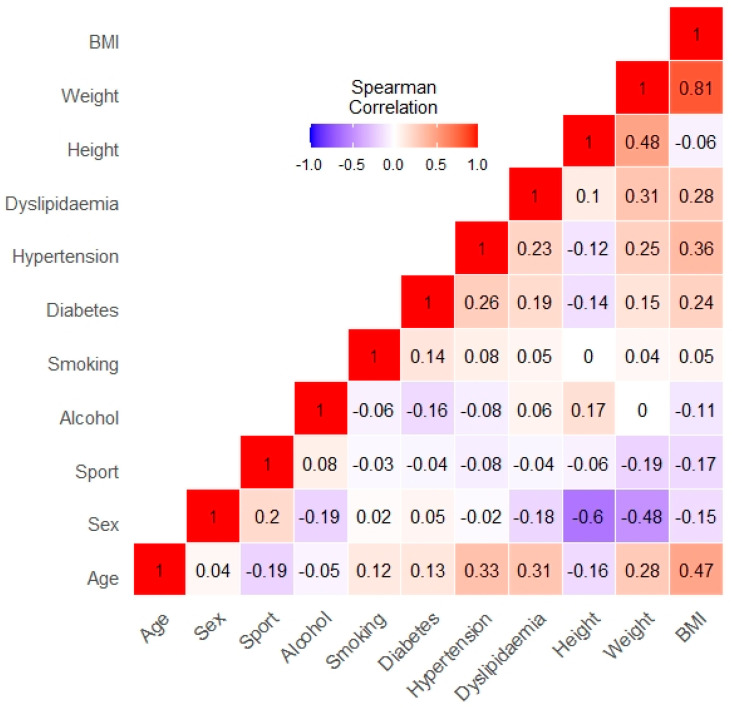
Spearman correlation table of the investigated risk factor and the measured characteristics of our population made with RStudio version 1.3.1093. BMI: body mass index.

We should note that a few of our environmental variables are correlated (Figure 3). Therefore, it is possible, that in a few cases we could not show significant effects, when in reality they may exist due to multicolinearity.

## 4. Discussion

To the best of our knowledge, this is the first twin study that measures the heritability of different morphological measurements of the basilar artery. Our findings demonstrated that the length and volume of the BA is genetically determined, but the role of unique environmental effects remain an important factor. Furthermore, the right VA curvature was only partly genetically influenced, but was greatly impacted by the unshared environment. The remaining measured anthropometric indices were affected by the shared and unshared environmental effects.

The vertebral artery difference (VAD) and hypoplasia are common anatomical variations that can be found on MRI scans [13]. VAD can increase the risk of cerebellar ischemic stroke [6,7]. The hemodynamic mechanism of the ischemic stroke in patients with VAD is not yet completely understood, but distal embolization, caused mainly by atherosclerosis seems to be a predisposing factor [13,14]. The smaller caliber size of the non-dominant VA may result in a lower mean flow and irregular wall shear stress, which increases the risk of prothrombotic or atherosclerotic processes leading to increased curvature, stenosis, or occlusion [14,15]. Hong et al. found that amongst posterior inferior cerebellar artery infarction patients, 72.7% of these lesions occurred on the non-dominant side of the VA [6]. Our results show that the V4 section of the VA on both sides was mainly influenced by shared and unshared environmental factors. In contrast, our research group previously (2017) demonstrated that the diameter of the V1 and V2 segments of the VA is moderately heritable [8]. This difference may arise from the fact that the V4 segment of the VA is more distal from the heart, increasing the effects of hemodynamic disturbances on the V4 segment [16]. However, our results also showed that the right side VA curvature was moderately heritable. This could be, in theory, explained with a lower flow on the right side due to it not directly branching off the aortic arch and thus having lower, inconsistent wall shear stress and flow velocities. Yuan et al. found that the asymmetry and variance of the V1-V2 segments are less common compared to the V4 segments [17]. Wake-Buck et al. identified the VA curvature and orientation at the two vertebral arteries’ confluence as important factors affecting blood flow velocities based on computational fluid dynamics modeling [3]. In utero factors such as differences in placentation or maternal (external) effects may also influence intrauterine neurogenesis, which may affect cerebral vascular development at the epigenetic level [18].

It has been shown that the uneven VA flow may cause curving and elongation of the BA [7]. The BA length and curvature (or bending) has also been linked to an increased risk of pontine and thalamic ischemic stroke [6,7]. The inner wall of the lower curve of the BA may be more thrombogenic due to the low wall shear stress [19]. Furthermore, the traction of the pontine perforating arterioles caused by the BA bending may also lead to infarction [20]. The BA curvature showed a high unshared environmental effect (83.6%; 95% CI: 0.633–1). These findings are in line with previous studies, which suggested that hemodynamic changes caused by lifestyle and comorbidities such as hypertension and diabetes along with VAD are the main cause of BA bending. Jeong et al. demonstrated that patients with deep pontine lacunar infarcts were older, had a bigger VAD, and measured higher BA angulations [21]. Zhu et al. found similar trends and an added history of hypertension, smoking, high homocysteine, high cholesterol, and type 2 diabetes as risk factors for the increased BA curvature [7]. This was in accordance with our findings, which showed that smoking affected the dimension of the BA, however diabetes, hypertension, and dyslipidemia did not correlate significantly. This could be either due to the fact that our population consisted of younger participants, or since our patients only consisted of asymptomatic individuals. Zhang et al. identified a BA curvature greater than 3.77 mm as an independent risk factor for pontine infarction [5]. Kwon et al. found that among the patients diagnosed with pontine infarcts, the BA volume was higher in paramedian pontine infarcts (*p* = 0.016), while the BA length was higher in lacunar pontine infarcts (*p* = 0.034) [22]. Our results showed that the BA length and volume were moderately heritable. This may be in part explained by the outcome of recent brain volume twin studies, in which Zhao et al. found high brainstem volume heritability [23]. Our hypothesis is that under normal anatomical circumstances, the basilar artery volume and length would increase along with an individual’s brainstem volume, but also be affected by hemodynamic disturbances caused by shared and unshared environmental factors. However, further studies are needed to elucidate if there is an overlap between the heritability of brainstem volume and BA length and volume phenotypes. The BA atherosclerosis has also been linked to an increased lesion volume in the subacute pontine infarction [24].

This study may draw attention to the background of the vertebrobasilar system’s morphological indices. Although the BA length and volume are moderately heritable, the BA bending and VAD, which are important risk factors for a posterior circulation stroke, are mainly influenced by shared and unshared environmental factors. It seems that patients with an increased BA curvature and/or VAD would benefit from a healthy lifestyle to decrease the effects of unhealthy unshared environmental factors. Treating the modifiable, known risk factors for large-vessel atherosclerosis, may decrease the risk of a posterior circulation stroke. The potential clinical relevance of this research is that it could play a role in the development of detecting intracranial hemodynamic disturbances and may help with neuro-interventional procedure planning.

Our study had five limitations. First, our twin population may not be an accurate representation of the general population. Second, we used TOF MR images that visualize the flow in the posterior intracranial circulation and not the anatomical properties of the vessels, which may produce artifacts that can influence our measurements. Third, since our study did not contain flow velocity measurements of the vertebral arteries, there was no proof of a weakened compensation of the non-dominant VA. Our monozygotic twin population was also significantly younger than our dizygotic and thus, the exposure to environmental factors may have been shorter in this group. Lastly, although the VA difference showed a higher monozygotic within-pair correlation, it did not fit the saturated model significantly. Consequently, no exact conclusion can be drawn from these findings except that the heritability of these parameters cannot be ruled out, and a larger population size is required to better understand this index.

## 5. Conclusions

In summary, the length and volume of the BA has a moderate genetical influence. However, most of the measured morphological indices were influenced by shared and unshared factors, which may highlight the complex hemodynamic background of the vertebrobasilar system. These findings support further collaborative initiatives to localize the specific genes involved in the vertebrobasilar system’s development and regulation.

## Figures and Tables

**Table 1:** Demographic, clinical characteristics, and vessel morphological measurements by zygosity.

	Total	MZ	DZ	*p*
Zigosity (*n* paires)	100	67	33	-
Age	51.66 (±14.96)	49.57 (±14.42)	56 (±15.23)	0.004 *
Sex (F:M)	132:67	90:44	43:23	-
Does weekly exercise	63.00%	68.65%	51.51%	0.02 *
Alcohol consumption once a week	54.00%	52.23%	59.09%	0.29
Ever Smoked	27.00%	28.35%	24.24%	0.60
Diagnosed with diabetes	8.50%	9.70%	6.06%	0.41
Diagnosed with hypertension	30.50%	30.60%	30.30%	0.95
Diagnosed with dyslipidaemia	24.50%	24.62%	24.24%	0.97
Height (cm)	167.95 (±9.12)	167.90 (±9.27)	168.05 (±8.86)	0.91
Weight (kg)	72.75 (±14.23)	72.52 (±14.80)	73.22 (±13.07)	0.74
BMI (kg/m^2^)	25.74 (±4.59)	25.56 (±4.61)	26.12 (±4.57)	0.41
Basilar artery length (mm)	24.08 (±4.41)	23.77 (±4.5)	24.73 (±4.18)	0.14
Basilar artery diameter (calculated mm)	3.42 (±0.57)	3.39 (±0.54)	3.49 (±0.61)	0.23
Basilar artery area (mm²)	9.43 (±3.02)	9.23 (±2.88)	9.84 (±3.26)	0.17
Basilar artery volume (mm³)	218.28 (±81.32)	210.47 (±79.24)	234.49 (±83.79)	0.05
Basilar artery curvature (mm)	2.57 (±2.29)	2.51 (±2.16)	2.68 (±2.54)	0.06
Basilar artery tortuosity (%)	6.37%	6.03%	7.08%	0.35
Basilar artery torsion (%)	11.29%	10.59%	12.57%	0.19
Left vertebral artery diameter (mm)	2.44(±0.72)	2.44 (±0.74)	2.45 (±0.69)	0.92
Right vertebral artery diameter (mm)	2.36 (±0.61)	2.36 (±0.56)	2.36 (±0.7)	0.91
Vertebral artery difference (mm)	0.75 (±0.62)	0.71 (±0.61)	0.82 (±0.66)	0.22
Left vertebral artery curvature (%)	7.79%	7.88%	7.61%	0.42
Right vertebral artery curvature (%)	8.10%	8.14%	8.04%	0.77
Left vertebral artery tortuosity (%)	11.77%	11.12%	13.10%	0.23
Right vertebral artery tortuosity (%)	11.57%	10.71%	13.35%	0.07
Left vertebral artery torsion (%)	12.34%	12.64%	11.73%	0.51
Right vertebral artery torsion (%)	12.62%	12.75%	12.35%	0.78

*: Indicates a significant difference between the mono- and dizygotic groups. BMI: body mass index; MZ: Monozygotic; DZ: Dizygotic.

**Table 2:** Age- and sex-adjusted parameter estimates for additive genetic (A), common environmental (C), and unique environmental influences (E) on the different measured parameters by structural equation modeling (95% confidence intervals). The most parsimonious, univariate structural equation model estimates are marked with * and highlighted as bold. R: Intrapair correlation; MZ: Monozygotic; DZ: Dizygotic; AIC: Akaike information criteria; BIC: Bayesian information criteria.

	Model	AIC	BIC	A	95% CI	C	95% CI	E	95% CI
Basilar artery length (mm)	A-C-E	1179.85	1166.67	0.63	0.457–0.752	0	0–0.445	0.37	0.248–0.543
*rMZ: 0.616 (0.434 0.749)*	A-E *	1177.11	1165.19	0.63	0.457–0.752			0.37	0.248–0.543
*rDZ: 0.288 (−0.048 0.566)*	C-E	1182.74	1170.82			0.503	0.331–0.641	0.497	0.359–0.669
Basilar artery area (mm²)	A-C-E	277.860	264.687	0.13	0–0.571	0.252	0–0.513	0.618	0.426–0.827
*rMZ: 0.361 (0.127 0.558)*	A-E	275.811	263.888	0.409	0.192–0.585			0.591	0.415–0.808
*rDZ: 0.346 (0.02 0.606)*	C-E *	275.271	263.347			0.354	0.168–0.516	0.646	0.484–0.832
Basilar artery volume (mm³)	A-C-E	395.808	382.634	0.601	0.255–0.732	0	0–0.292	0.399	0.268–0.576
*rMZ: 0.646 (0.474 0.771)*	A-E *	393.073	381.149	0.601	0.424–0.732			0.399	0.268–0.576
*rDZ: 0.016 (−0.316 0.347)*	C-E	401.440	389.516			0.452	0.281–0.596	0.548	0.404–0.719
Basilar artery curvature (mm)	A-C-E	1244.914	1231.741	0	0–0.371	0.164	0–0.347	0.836	0.653–1
*rMZ: 0.117 (−0.133 0.354)*	A-E	1242.810	1230.887	0.174	0–0.388			0.826	0.612–1
*rDZ: 0.231 (−0.098 0.514)*	C-E *	1242.179	1230.255			0.164	0–0.347	0.836	0.653–1
Basilar artery tortuosity (%)	A-C-E	611.919	598.746	0.099	0–0.627	0.395	0–0.618	0.507	0.345–0.699
*rMZ: 0.492 (0.275 0.663)*	A-E	610.983	599.059	0.515	0.32–0.664			0.485	0.336–0.68
*rDZ: 0.464 (0.162 0.686)*	C-E *	609.296	597.373			0.475	0.297–0.621	0.525	0.379–0.703
Basilar artery torsion (%)	A-C-E	-201.823	-214.996	0	0–0.239	0	0–0.196	1	0.761–1
*rMZ: −0.003 (−0.246 0.241)*	C-E	-204.558	-216.482			0	0–0.196	1	0.804–1
*rDZ: −0.016 (−0.345 0.317)*	E *	-207.233	-217.967					1	1
Left vertebral artery diameter (mm)	A-C-E	453.901	440.727	0.003	0–0.436	0.229	0–0.412	0.768	0.563–0.965
*rMZ: 0.255 (0.011 0.473)*	A-E	451.515	439.592	0.249	0.03–0.445			0.751	0.555–0.97
*rDZ: 0.213 (−0.124 0.506)*	C-E *	451.166	439.242			0.232	0.035–0.412	0.768	0.588–0.965
Right vertebral artery diameter (mm)	A-C-E	151.110	137.936	0.181	0–0.601	0.257	0–0.559	0.562	0.39–0.765
*rMZ: 0.454 (0.236 0.629)*	A-E	148.959	137.035	0.453	0.254–0.612			0.547	0.388–0.746
*rDZ: 0.339 (0.016 0.598)*	C-E *	148.656	136.732			0.41	0.228–0.564	0.59	0.436–0.772
Vertebral artery difference (mm)	A-C-E	588.849	575.675	0.23	0–0.444	0	0–0.29	0.77	0.556–1
*rMZ: 0.306 (0.064 0.514)*	A-E *	586.114	574.190	0.23	0–0.444			0.77	0.556–1
*rDZ: −0.089 (−0.405 0.248)*	C-E	587.741	575.817			0.144	0–0.335	0.856	0.665–1
Left vertebral artery curvature (%)	A-C-E	57.607	44.434	0.064	0–0.522	0.274	0–0.494	0.662	0.474–0.858
*rMZ: 0.349 (0.116 0.547)*	A-E	55.443	43.519	0.358	0.148–0.535			0.642	0.465–0.852
*rDZ: 0.284 (−0.061 0.567)*	C-E *	54.900	42.976			0.329	0.139–0.495	0.671	0.505–0.861
Right vertebral artery curvature (%)	A-C-E	48.395	35.221	0.216	0–0.428	0	0–0.292	0.784	0.572–1
*rMZ: 0.27 (0.027 0.483)*	A-E *	45.659	33.735	0.216	0–0.428			0.784	0.572–1
*rDZ: −0.066 (−0.381 0.263)*	C-E	46.984	35.060			0.141	0–0.327	0.859	0.673–1
Left vertebral artery tortuosity (%)	A-C-E	509.755	496.581	0.339	0–0.635	0.124	0–0.522	0.536	0.365–0.769
*rMZ: 0.409 (0.185 0.594)*	A-E *	507.207	495.284	0.476	0.263–0.638			0.524	0.362–0.737
*rDZ: 0.349 (0.018 0.611)*	C-E	508.125	496.202			0.386	0.2–0.545	0.614	0.455–0.8
Right vertebral artery tortuosity (%)	A-C-E	490.434	477.260	0.557	0.043–0.711	0	0–0.391	0.443	0.289–0.659
*rMZ: 0.477 (0.248 0.655)*	A-E *	487.698	475.775	0.557	0.341–0.711			0.443	0.289–0.659
*rDZ: 0.281 (−0.048 0.556)*	C-E	492.061	480.138			0.385	0.199–0.544	0.615	0.456–0.801
Left vertebral artery torsion (%)	A-C-E	-183.109	-196.283	0.057	0–0.271	0	0–0.23	0.943	0.729–1
*rMZ: 0.078 (−0.167 0.316)*	C-E	-185.738	-197.662			0.039	0–0.233	0.961	0.767–1
*rDZ: −0.06 (−0.395 0.292)*	E *	-188.263	-198.997					1	1
Right vertebral artery torsion (%)	A-C-E	-165.418	-178.592	0	0–0.168	0	0–0.186	1	0.814–1
*rMZ: −0.1 (−0.336 0.149)*	C-E	-168.154	-180.078			0	0–0.186	1	0.814–1
*rDZ: 0.225 (−0.104 0.512)*	E *	-170.829	-181.563					1	1

**Table 3:** Significant differences between the variables after correcting the final ACE models for age, sex, sport activity, alcohol, smoking, diabetes, hypertension, dyslipidemia, height, weight, and BMI.

	-2LL_Base	-2LL_Reduced	*p*
Basilar area	242.4058	280.1872	<0.001
Basilar volume	360.3536	401.4033	<0.001
Basilar diameter	292.5449	330.3450	<0.001
Left VA curvature	22.15288	37.39575	0.05
Right VA curvature	12.94010	39.96847	<0.001
Left VA diameter	418.4466	435.5442	0.03
Basilar length	1144.393	1165.349	0.01

-2LL_base: -2*Loglikelihood of the model containing all the variables, -2LL_reduced: -2*Loglikelihood of the model containing only age and sex, *p*: *P*-value of the likelihood ratio test; VA: vertebral artery.

**Table 4:** Smoking and height were consistent across similar variables and showed a significant effect on the dimensions of the basilar artery, while only smoking affects the vertebral artery.

Model	Smoking (Binary)	Height (cm)
Left VA diameter	0.22 (0.002, 0.435)	Not significant
Basilar diameter	0.212 (0.049, 0.375)	0.025 (0.012, 0.037)
Basilar volume	Not significant	0.03 (0.015, 0.045)
Basilar area	0.188 (0.043, 0.332)	0.022 (0.011, 0.033)

95% confidence intervals in the parenthesis.
